# Regulation of nitrite resistance of the cytochrome *cbb*_3_ oxidase by cytochrome *c* ScyA in *Shewanella oneidensis*

**DOI:** 10.1002/mbo3.224

**Published:** 2014-11-22

**Authors:** Jianhua Yin, Miao Jin, Haiyan Zhang, Lili Ju, Lili Zhang, Haichun Gao

**Affiliations:** 1 Institute of Microbiology and College of Life Sciences, Zhejiang UniversityHangzhou, Zhejiang, 310058, China; 2 College of Life Sciences, Tarim UniversityAlar, Xinjiang, China

**Keywords:** Cytochrome *c*, nitrite resistance, Shewanella, the cytochrome *cbb*_3_ oxidase

## Abstract

Cytochrome *c* proteins, as enzymes to exchange electrons with substrates or as pure electron carriers to shuttle electrons, play vital roles in bacterial respiration and photosynthesis. In *Shewanella oneidensis*, a research model for the respiratory diversity, at least 42 *c*-type cytochromes are predicted to be encoded in the genome and are regarded to be the foundation of its highly branched electron transport pathways. However, only a small number of *c*-type cytochromes have been extensively studied. In this study, we identify soluble cytochrome *c* ScyA as an important factor influencing the nitrite resistance of a strain devoid of the *bd* oxidase by utilizing a newly developed transposon mutagenesis vector, which enables overexpression of the gene(s) downstream of the insertion site. We show that when in overabundance ScyA facilitates growth against nitrite inhibition by enhancing nitrite resistance of the *cbb*_3_ oxidase. Based on the data presented in this study, we suggest two possible mechanisms underlying the observed effect of ScyA: (1) ScyA increases electron flow to the *cbb*_3_ oxidase; (2) ScyA promotes nitrite resistance of the *cbb*_3_ oxidase, possibly by direct interaction.

## Introduction

*Shewanella oneidensis*, a facultative Gram-negative anaerobe, is renowned for its remarkable respiration versatility (Fredrickson et al. 2008). Linked to this unique characteristic is a high content of *c*-type cytochromes (Heidelberg et al. 2002; Meyer et al. 2004; Romine et al. 2008; Gao et al. 2010a), which are characterized by the covalent attachment of the prosthetic cofactor (the heme group) to the protein polypeptide at the cysteines within the signature heme *c* binding motif (HBM) CX_2_CH although exceptions exist (Kranz et al. 2009). These proteins, existing as membrane-bound or soluble in the periplasm, play vital roles in bacterial respiration as enzymes to exchange electrons with the bound substrates or as pure electron carriers to shuttle electrons.

Many of the functionally defined *S. oneidensis c*-type cytochromes are terminal reductases as their corresponding deletion mutants display distinguishable phenotypes, such as NrfA (nitrite reductase), NapB (small subunit of nitrate reductase), FccA (fumarate reductase), and DmsE (subunit of dimethyl sulfoxide [DMSO] reductase), to name a few (Tsapin et al. 2001; Gralnick et al. 2006; Gao et al. 2009). Furthermore, those involved in the respiration of insoluble electron acceptors (EAs), such as metal oxide, are relatively better understood because the subject has been under intensive investigation in the microorganism since its isolation (Fredrickson et al. 2008; Richardson et al. 2012; Richter et al. 2012). Nevertheless, a large fraction of the entire *c*-type cytochrome pool remains poorly defined since the loss of many of these *c*-type cytochromes individually does not result in distinct phenotypes (Bretschger et al. 2007; Gao et al. 2010a; Jin et al. 2013). Multiple mechanisms may underlie this phenomenon. First, a number of *c*-type cytochromes are not produced to a level of physiological relevance under the experimental conditions revealed in both transcriptomic and proteomic analyses, of which the cytochrome *caa*_3_ oxidase serves as a good example (Nissen et al. 2012; Jin et al. 2013; Zhou et al. 2013; Le Laz et al. 2014). Second, there exists the promiscuity of *c*-type cytochromes given that a large number of proteins sharing similar features coexist in the proteome, especially in the case of small periplasmic *c*-type cytochromes (Fonseca et al. 2013; Fu et al. 2014). Moreover, certain terminal reductases may be able to reduce multiple substrates, functionally overlapping substrate-specific reductases. For instance, in vitro analyses reveal that Otr, octaheme tetrathionate reductase (SO4144), is able to reduce nitrite and hydroxylamine and NrfA can carry out the reduction of sulfite to sulfide, although in both cases whether these substrates are the real physiological substrates is yet determined (Atkinson et al. 2007; Lukat et al. 2008). The situation is further complicated by the finding that *c*-type cytochromes can be functionally replaced by noncytochrome proteins (Cordova et al. 2011). In this case, an NrfD/PsrC-like protein (SirD), in association with an iron–sulfur redox partner (SirC), is in large part able to substitute CymA, a *c*-type cytochrome that couples to multiple terminal reductases and periplasmic electron transfer proteins (Marritt et al. 2012).

It is well known that nitrite is toxic to bacterial cells as it has been used as preservative in meat products for centuries. Although nitrite (≤2 mmol/L) can be used as sole EA to support growth of *S. oneidensis*, it imposes a greatly negative impact on viability and growth at higher concentrations (Gao et al. 2009; Dong et al. 2012; Fu et al. 2013; Zhang et al. 2013). The *S. oneidensis* genome encodes three terminal oxidases: a *bd*-type quinol oxidase encoding by the *cydABX* genes and two heme–copper oxidases (HCOs), a *caa*_3_-type and a *cbb*_3_-type encoded by the *SO4606-9* and *ccoNOQP* genes, respectively (Heidelberg et al. 2002; Buschmann et al. 2010; Chen et al. 2014). According to their predicted affinity for molecular oxygen, the *caa*_3_-HCO and *cbb*_3_-HCO should operate under aerobic and microaerobic conditions, respectively. Interestingly, the *cbb*_3_-HCO is abundant in membranes under both aerobic and microaerobic conditions, whereas the *caa*_3_-HCO is not present (Zhou et al. 2013; Le Laz et al. 2014). The *bd* oxidase, like its *Escherichia coli* counterpart, plays a role in oxygen respiration under microaerobic but not aerobic conditions, and probably more importantly, confers nitrite and nitric oxide (NO) resistance to *S*. *oneidensis* (Borisov et al. 2011; Fu et al. 2013; Zhang et al. 2013; Chen et al. 2014).

Recently, we identified the cytochrome *bc*_1_ complex as a functional replacement of CymA in respiration of nitrate and nitrite by utilizing a transposon-based screening (Fu et al. 2014). Given that a large number of proteins remain functionally undefined, we reason that proteins capable of enhancing nitrite resistance, in absence or excess, may be present in *S. oneidensis*. In this study, we tested this hypothesis by screening the genome for genes encoding such proteins with a newly developed transposon vector, which contains a strong promoter embedded within the transposable segment. We found that overproduction of ScyA, a periplasmic cytochrome *c*, elevated nitrite resistance of a mutant lacking the *bd* oxidase. Subsequent analysis revealed that this effect was associated with the *cbb*_3_-HCO. We further showed that ScyA in excess did not affect the abundance of the *cbb*_3_-HCO. Instead, the protein when overproduced enhanced the resistance of the *cbb*_3_-HCO to nitrite either by increasing electron flow to the enzyme or by modulating biochemical properties of the enzyme via direct contact.

## Experimental Procedures

### Bacterial strains, plasmids, polymerase chain reaction primers, and culture conditions

Bacterial strains and plasmids used in this study are listed in Table1. Sequences of the primers used for generating polymerase chain reaction (PCR) products are available upon request. Chemicals were acquired from Sigma unless otherwise noted. For genetic manipulation purpose, *E. coli* and *S. oneidensis* strains were grown in Luria–Bertani (LB; Difco, Beijing, China) medium at 37°C and 30°C, respectively. When needed, growth medium was supplemented with chemicals at the following concentrations: 2,6-diaminopimelic acid, 0.3 mmol/L; ampicillin, 50 *μ*g/mL; kanamycin, 50 *μ*g/mL; and gentamycin, 15 *μ*g/mL.

**Table 1:** Strains and plasmids used in this study

Strain or plasmid	Description	Reference or source
*Escherichia coli* strains		
DH5*α*	Host strain for cloning	Lab stock
WM3064	Donor strain for conjugation; Δ*dapA*	W. Metcalf, UIUC
*Shewanella oneidensis* strains		
MR-1	Wild-type	ATCC 700550
HG0264	Δ*scyA* derived from MR-1	Jin et al. (2013)
HG0608-10	Δ*pet* derived from MR-1	Fu et al. (2014)
HG0624	Δ*crp* derived from MR-1	Gao et al. (2010b)
HG2364-1	Δ*cco* derived from MR-1	This study
HG3286-4	Δ*cyd* derived from MR-1	This study
HG3980	Δ*nrfA* derived from MR-1	Gao et al. (2009)
HG3896	Δ*ompS38* derived from MR-1	This study
HG4591	Δ*cymA* derived from MR-1	Gao et al. (2009)
HGCCO-CYD	Δ*cco*Δ*cyd* derived from MR-1	This study
HGSCYA-CYD	Δ*scyA*Δ*cyd* derived from MR-1	This study
HGCYD-CYMA	Δ*cyd*Δ*cymA* derived from MR-1	This study
HGCYD-PET	Δ*cyd*Δ*pet* derived from MR-1	This study
HGCYD-NRFA	Δ*cyd*Δ*nrfA* derived from MR-1	This study
Plasmids		
pHGM01	Ap^R^, Gm^R^, CM^R^, suicide vector	Jin et al. (2013)
pHG101	Promoterless broad-host Km^R^ vector	Wu et al. (2011)
pHG102	pHG101 containing the *S. oneidensis arcA* promoter	Wu et al. (2011)
pFAC	Mariner-based transposon vector	Wong and Mekalanos (2000)
pHGC01	Integrative vector for complementation	Fu et al. (2013)
pTP327	Multicopy *E. coli lacZ* reporter vector	Gao et al. (2010b)
pHGEI01	Integrative *E. coli lacZ* reporter vector	Fu et al. (2014)
pHGE-P*tac*	Broad-host IPTG-inducible expression vector	Luo et al. (2013)
pHGT01	Promoter-embedded Mariner-based transposon vector	This study
pHGE-ScyA-His_6_	His-tagged ScyA expression vector	This study

IPTG, isopropyl *β*-d-1-thiogalactopyranoside.

M1 defined medium containing 0.02% (w/v) of vitamin-free casamino acids and 15 mmol/L lactate as electron donor was used to evaluate impacts of mutations of interest on aerobic growth as described before (Gao et al. 2008). Growth of the deletion strains was determined by recording the optical density of cultures at 600 nm (OD_600_) with the wild type as the control. Fresh media were inoculated with exponential phase cultures to ∼0.01 of OD_600_ and shaken at 200 rpm 30°C. For nitrite sensitivity purpose, LB medium was used.

### In-frame deletion mutagenesis and complementation

In-frame deletion strains were constructed according to the *att*-based Fusion PCR method described previously (Jin et al. 2013). In brief, two fragments flanking the targeted gene were amplified with primers containing *attB* and the gene-specific sequence, and then joined by a second round of PCR. The resulting fusion fragment was introduced into plasmid pHGM01 by site-specific recombination using the BP Clonase (Invitrogen, Shanghai, China) and maintained in *E. coli* WM3064. The resulting mutagenesis vector was then transferred from *E. coli* into *S. oneidensis* by conjugation. Integration of the mutagenesis construct into the chromosome was selected by gentamycin resistance and confirmed by PCR. Verified transconjugants were grown in LB broth in the absence of NaCl and plated on LB supplemented with 10% sucrose. Gentamycin-sensitive and sucrose-resistant colonies were screened by PCR for deletion of the targeted gene. The deletion mutations were then verified by sequencing.

Plasmids pHG101 and pHG102 were used in genetic complementation of mutants and overexpression of gene of interest (Wu et al. 2011). For complementation of genes adjacent to their promoters, a fragment containing the gene of interest and its native promoter was generated by PCR and cloned into pHG101. Otherwise, the gene of interest was amplified and inserted into MCS of pHG102 under the control of the *S. oneidensis arcA* promoter, which is constitutively active (Gao et al. 2010b). To single-copy complementation, pHGC01 was used as described previously (Fu et al. 2014). The resulting complementation vector was transferred into its relevant mutant strains via conjugation and its presence was confirmed by plasmid purification and restriction enzyme digestion.

### Transposon mutagenesis

A random mutagenesis vector with an embedded robust promoter was developed in this study. We previously noticed that pFAC, a mariner-based random mutagenesis vector (Wong and Mekalanos 2000), was substantially lower in the transfer rate via conjugation from *E. coli* to *S. oneidensis* than pHGC01 (Fu et al. 2013). To increase the transfer efficiency of the new vector derived from pFAC, we amplified the *R6K* replicon and the *mob* gene with pHGC01 as the template and used the resulting fragment to replace the DNA sequence of pFAC beyond that for IS elements, gentamycin-resistant gene, and *Himar1* transposase. Subsequently, we replaced P_*tn*_, the promoter embedded in the transposable sequence of pFAC, with a promoter that is constitutively robust under most conditions so as to overexpress the gene after the insertion. An array of microarray data from our prior studies were examined and three genes from single gene operons with high signal intensities under all conditions were chosen for further validation (Gao et al. 2004, 2006, 2008, 2009; Jiang et al. 2014). These include *SO2593*, *SO3896*, and *cymA* (SO4591), encoding a hypothetical protein, an outer membrane porin (Maier and Myers 2004), and a well-studied tetraheme cytochrome *c*, respectively.

An analysis of sequences upstream of these three genes using Neural Network Promoter Prediction revealed that their predicted transcription starting sites of most confidence were within 350 bp relative to the translation initiation codon (Reese 2001). Consequently, for each gene a fragment covering 400 bp of sequence upstream of the coding region was amplified and cloned into a *lacZ* reporter system for the quantification of the promoter activity. As shown in Figure S2A, within multiple-copy pTP327 all of three promoters were much more robust than P_*tn*_ and P_*arcA*_, a constitutively active promoter for global regulator ArcA (Gao et al. 2010b). Among them, P_*so3896*_ appeared to be particularly strong, approximately 400% over other two promoters. To further confirm this, we transferred these promoters to an integrative *lacZ* reporter system pHGEI01 such that their activities can be assessed in a single copy (Fu et al. 2014). Once transferred into *S. oneidensis* strains, pHGEI01 containing promoter of interest integrates into the chromosome and the antibiotic marker is then removed by an established approach (Fu et al. 2013). While these promoters in a single copy drove expression of *β*-galactosidase substantially less effectively compared to them in multiple copies, they were consistently more active than P_*tn*_ and P_*arcA*_ (Fig. S2A). As P_*so3896*_ is the strongest, we further examined the expression of the gene by analyzing the abundance of its product in the outer membrane. The insoluble membrane fractions from cells cultured in normal and high-osmolarity media were harvested and applied to sodium dodecyl sulfate–polyacrylamide gel electrophoresis (SDS-PAGE) for separation (Fig. S2B). SO3896, validated by the loss of the band in an *SO3896* mutant, was apparently one of the major outer membrane proteins under test conditions, confirming that P_*so3896*_ is robust and relatively constitutive. We, therefore, chose it for the subsequent construction. To drive transcription of genes immediately downstream of the insertion, P_*so3896*_ was placed outwards within the transposable fragment using conventional cloning approaches, resulting in pHGT01 (Fig. S2C). By transferring pHGT01 into the Δ*cyd* strain by conjugation, a random mutation library was constructed and nitrite resistance suppressor strains were selected with gentamycin and 3 mmol/L nitrite. According to the number of colonies on the control plates (Gm^+^Nitrite^−^), the transposon library was estimated to contain more than 100,000 individual insertion mutants. Colonies, obtained from Gm^+^Nitrite^+^ plates, were subjected to the mapping of the transposon insertion sites using the arbitrary PCR (Das et al. 2005).

### SDS-PAGE analysis of outer membrane proteins

Envelope fractions were prepared according to an established method with modifications (Slauch and Silhavy 1989). Briefly, cultures of mid-log phase (∼0.4 of OD_600_) were normalized to the lowest OD to allow for comparison of outer membrane protein quantities across strain backgrounds. Normalized cultures were pelleted, washed once in 30 mmol/L Tris-HCl (pH 8.1), and pelleted again at 3800*g*. Cell pellets were then resuspended in 30 mmol/L Tris-HCl–20% sucrose buffer (500 *μ*L), followed by the addition of 20 *μ*L of 20-mg/mL lysozyme–0.1 mmol/L Ethylenediaminetetraacetic acid (EDTA) (pH 7.3), and incubated on ice for 30 min. Following lysozyme treatment, 1 mL of 3 mmol/L ETDA (pH 7.3) was added and the resulting extract was disrupted with a single 20-s pulse using a microtip sonicator (Haishu Instrument, Ningbo, China). A 1.5-mL fraction of the extract was then centrifuged at 16,000*g* for 60 min. Envelope fractions were collected as centrifuged precipitate and resuspended in 30 *μ*L of Laemmli SDS sample buffer. After boiled for 5 min, 15-*μ*L samples were subjected to SDS-PAGE (10% acrylamide, 6 mol/L urea, 1% SDS).

### Expression and purification of *S. oneidensis* ScyA

An isopropyl *β*-d-1-thiogalactopyranoside (IPTG) inducible vector, pHGE-P*tac*, was used for expressing recombinant ScyA with 6x His-tag fused to its C-terminus in *S. oneidensis* (Luo et al. 2013). Cells were grown to late-exponential phase (∼0.8 of OD_600_), induced with 0.5 mmol/L IPTG for 2 h, collected by centrifugation, resuspended in lysis buffer (50 mmol/L Tris/HCl, pH 7.5, 200 mmol/L NaCl, 1 mmol/L MgCl_2_, 10 mmol/L *β*-mercaptoethanol, 1 mmol/L phenylmethanesulfonylfluoride (PMSF), 5 mg/mL DNase I), and broken by passage twice through a French press (10,000 psi). The soluble recombinant ScyA protein was purified using a talon resin column (BD Biosciences, Beijing, China) according to the manufacturer's instructions. The protein concentration in the collected samples here, and all other samples mentioned in this study, was determined using a Bradford assay with bovine serum albumin (BSA) as a standard (Bio-Rad, Shanghai, China). Sample purity was checked by SDS-PAGE.

### Site-directed mutagenesis

Plasmid pHG101-*scyA-*His_6_ was used as the template for site-directed mutagenesis with a QuikChange II XL site-directed mutagenesis kit (Stratagene, Santa Clara, CA, USA) as described previously (Sun et al. 2013).

### Expression analyses

The activity of promoters of interest was assessed using both multiple-copy and single-copy integrative *lacZ* reporter systems as described previously (Gao et al. 2010b; Fu et al. 2014). Based on promoter prediction, transcription start sites of all high-confident promoters are located within −350 relative to the translational initiation codon. A fragment covering the sequence upstream of each operon tested from −400 to +1 was then amplified and cloned into the reporter vectors pTP327 and pHGEI01, verified by sequencing, and the correct plasmid was then transferred into *S. oneidensis* strains by conjugation. Cells grown to the mid-exponential phase under experimental settings were collected and *β*-galactosidase activity was performed with an assay kit as described previously (Wu et al. 2011).

### Immunoblotting assay

Rabbit polyclonal antibodies against *S. oneidensis* ScyA were prepared in accordance with standard protocols by Genscript (Nanjing, China) and used for the immunoblotting analyses. Pellets of mid-log phase cells were washed once with phosphate-buffered saline (PBS), and resuspended in 2x Laemmli SDS sample buffer. After boiled for 5 min, samples were loaded onto 10% SDS-PAGE and either stained with Coomassie Brilliant Blue or electrophoretically transferred to polyvinylidene difluoride (PVDF) according to the manufacturer's instructions (Bio-Rad). Processing of the immunoblots was performed essentially as described previously (Dong et al. 2012). For estimation of relative abundance of proteins in the gel, band intensity was measured using ImageJ software (NIH).

### Cytochrome oxidase activity assay

Visual analysis of the *cbb*_3_-HCO activity was done by staining colonies with the agents for the Nadi Assay. Nadi reactions were carried out by the addition of *α*-naphthol and *N′,N′*-dimethyl-p-phenylenediamine (DMPD) on LB agar plates (Marrs and Gest 1973). Colonies were timed for formation of the indophenol blue.

Solubilized membranes were prepared for quantitative analysis of the cytochrome oxidase activity as described previously (Chen et al. 2014). In brief, cell pellets were resuspended in 20 mmol/L Tris-HCl (pH 7.6) supplemented with DNase I and protease inhibitors and disrupted by French pressure. After debris and unbroken cells removing, the membranes were pelleted by ultracentrifugation for 1 h at 230,000*g* at 4°C and subsequently resuspended in 20 mmol/L Tris-HCl pH 7.6 with 5% glycerol to a protein concentration of 10 mg/mL. Solubilization was performed with *n*-dodecyl *β*-d-maltoside (DDM) to a final concentration of 1% (w/v) on a rotary tube mixer for 2 h at 4°C. The DDM-solubilized membranes were obtained by collecting the supernatant after ultracentrifuging for 1 h at 230,000*g* at 4°C. The cytochrome oxidase activity was assayed as a measure of oxygen consumption rates using an OxyGraph oxygen electrode (Hansatech, Norfolk, UK) using either ubiquinol-1 or *N*,*N*,*N*′,*N*′-tetramethyl-p-phenylenediamine dihydrochloride (TMPD) as electron donor according to the methods described previously (Mason et al. 2009; Chen et al. 2014; Le Laz et al. 2014; Xie et al. 2014). The IC_50_ values of the cytochrome *bd* and *cbb*_3_-HCO for nitrite were obtained from plots of rates against nitrite concentrations.

### Heme Staining

Late-log phase cultures (∼0.8 of OD_600_) were pelleted by centrifugation at 5000*g* for 10 min. Heme staining of proteins from an aliquot of the pellet separated by SDS-PAGE (12.5%) was carried out with 3,3′,5,5′-tetramethylbenzidine (TMBZ) as described elsewhere (Thomas et al. 1976).

### Nitrite sensitivity assay and concentration determination

Cells of *S. oneidensis* strains grown to an OD_600_ of ∼0.4 were adjusted to approximately 10^7^ CFUs/mL, followed by 10-fold serial dilutions. Ten microliter of each dilution was spotted onto LB plates containing nitrite of varying concentrations. The plates were incubated at 30°C before being read. The assays were repeated at least three times with similar results. Concentrations of nitrite were determined by Ion Chromatography (IC) analysis (Gao et al. 2009).

### Statistical analyses

Values are presented as means ± SD (standard deviation). Student's *t*-test was performed with statistical significance set at the 0.05 confidence level.

## Results

### Screening for genes relevant to nitrite resistance in *S. oneidensis*

Our prior studies demonstrated that loss of the cytochrome *bd* oxidase (Δ*cyd*) results in a hypersensitive phenotype to nitrite (Fu et al. 2013; Zhang et al. 2013). As oxygen is the EA for growth under the experimental condition, this observation suggests that the cytochrome *bd* oxidase rather than other oxidases confers nitrite resistance to *S. oneidensis*, a notion supported by biochemical analyses of *E. coli* oxidases (Mason et al. 2009). *S. oneidensis* hosts a large number of electron transport proteins, some of which can enhance the activities of certain terminal reductases by offering additional electron transport pathways (Gao et al. 2009; Cordova et al. 2011; Fonseca et al. 2013; Fu et al. 2014). Moreover, although extensively studied, *S. oneidensis* remains poorly understood, having ∼40% of the proteome labeled as “functionally unknown” or “hypothetical”, a pool of candidates involved in nitrite resistance. Based on these, we reasoned that we could identify genes that play a role in nitrite resistance by either annulling or enhancing their expression.

Plasmid pFAC, a mariner-based transposon vector widely used for the construction of random insertion libraries in various bacteria, contains a promoter (P_*tn*_) embedded in the transposable sequence (Wong and Mekalanos 2000; Fu et al. 2013). However, in the presence of 3 mmol/L nitrite, we failed to obtain any colonies from a transposon insertion library derived from the Δ*cyd* strain. This is likely due to the fact that P_*tn*_ is intrinsically a rather weak promoter and thus unable to elevate transcription of gene of interest sufficiently high for conferring an evident increase in the resistance to nitrite (Fu et al. 2013). We therefore developed a new vector, pHGT01, by replacing i) P_*tn*_ with the *SO3896* promoter that is constitutively robust so that substantial overexpression of genes after the insertion can be achieved and ii) the sequence for replication and conjugation with the *R6K* replicon and the *mob* gene which is more efficient, as detailed in Experimental procedures. The *SO3896* gene encodes a mature porin polypeptide of 37.7 kDa (39.9 kDa before the signal peptide cleavage), and we therefore name the porin as OmpS38 (outer membrane porin of *Shewanella*, 38K).

**Figure 1:**
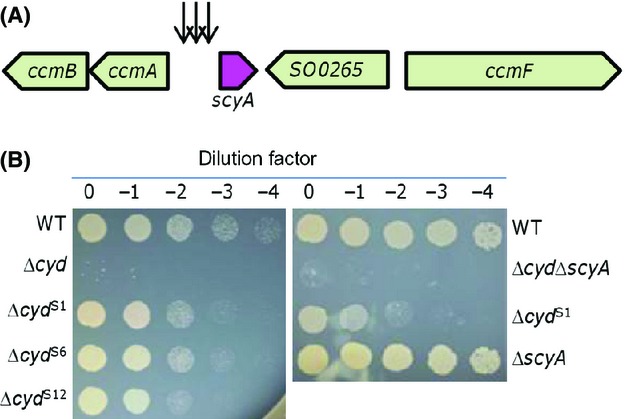
Transposon screens and terminal phenotypes. (A) Schematics indicating the approximate locations of the transposon insertions. Arrows represent transposon insertion points. (B) Nitrite sensitivity of representative Δ*cyd* suppressors isolated in the screen. Densities of mid-log phase cultures were normalized, 10-fold serial dilutions were prepared, and 5 *μ*L of each dilution was spotted onto LB containing 3 mmo/L nitrite. Experiments were repeated at least three times and similar results were obtained. LB, Luria–Bertani.

With pHGT01 we made attempts to identify genes involved in the nitrite resistance of *S. oneidensis* when expressed at substantially elevated levels. The vector was transferred into the Δ*cyd* strain by conjugation to construct a random mutation library. A total of ∼100,000 individual insertion mutants, estimated by colony-forming units (CFUs) on the nitrite-free control plates, were screened and 12 colonies were obtained on the plates supplemented with 3 mmol/L nitrite. Insertion sites of these 12 mutants were exclusively mapped to the region upstream of the *scyA* gene, which is flanked by genes encoding the cytochrome *c* maturation (Ccm) system (Jin et al. 2013) (Fig.1A). All colonies were orange-colored and displayed aerobic growth comparable to that of both the wild-type and Δ*cyd* strains (data not shown), ruling out a possibility that the Ccm system is damaged (Jin et al. 2013). A nitrite susceptibility assay confirmed that the nitrite resistance of these suppressors of the Δ*cyd* strain increased significantly compared to their parental strain (Fig.1B). However, they were still substantially more sensitive than the wild type, supporting the notion that the *bd* oxidase dictates the resistance (Fu et al. 2013). Given that all of the insertion sites are located within the intergenic rather than coding sequences, it is conceivable that enhanced expression of the *scyA* gene resulting from transposon insertion accounts for the elevated resistance of the Δ*cyd* strain to nitrite. To rule out the possibility that the increased resistance results from disruption of the *scyA* gene, we examined the nitrite susceptibility of strains lacking the *scyA* gene in either the wild-type or *cyd*^−^ background. Abilities of both Δ*scyA* and Δ*cyd*Δ*scyA* strains to respire on a variety of EAs, including oxygen, were similar to that of the wild type (data not shown). With respect to nitrite resistance, the Δ*scyA* and Δ*cyd*Δ*scyA* strains displayed a capacity indistinguishable from those of the wild-type and Δ*cyd* strains, respectively (Fig.1B), indicating that the loss of ScyA does not impair the nitrite resistance of *S. oneidensis*.

### Overexpression of the scyA gene increases nitrite resistance

ScyA is a small monoheme cytochrome *c*, which has been suggested to be one of the most abundant periplasmic proteins found in cells grown under various conditions (Meyer et al. 2004; Rosenbaum et al. 2012). Up to date, the only verified role for ScyA has been to function as a mediator of electron transport between CymA and CcpA, a periplasmic diheme *c*-type cytochrome peroxidase (Schütz et al. 2011; Fonseca et al. 2013).

**Figure 2:**
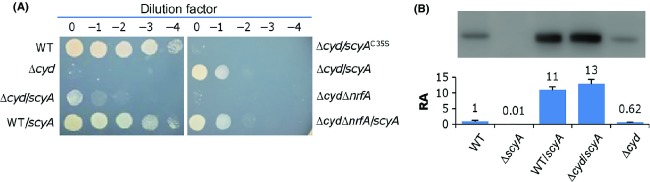
ScyA in excess increases nitrite resistance of the Δ*cyd* strain. All strains without overexpressing ScyA carried empty vectors. The assays were repeated at least three times and similar results were obtained. (A) Nitrite sensitivity of various *Shewanella oneidensis* strains as described in (B). ScyA^C^^35S^ is the a ScyA mutant whose the heme-binding site CTVCH was mutated to CTVSH. (B) Western blotting analysis for ScyA. To determine the extent of ScyA overproduction in the cultures from (A), extracts were prepared from cells grown to an OD_600_ of ∼0.8 under aerobic conditions. Immunoblot analysis was performed with antibodies against ScyA. Relative abundance (RA) of ScyA in these strains was estimated using software Image J. The values are the mean ± SD (error bars) (*n *= 4).

To provide direct evidence that overproduction of ScyA confers nitrite resistance to the Δ*cyd* strain, we cloned the *scyA* gene with its native promoter into pHG101 and introduced the resulting plasmid pHG101-P_*scyA*_-*scyA* into the wild-type and Δ*cyd* strains. Reportedly, this multiple-copy plasmid enables overproduction by approximately 5- to 15-fold (Wu et al. 2011; Dong et al. 2012; Fu et al. 2013; Sun et al. 2014). Although the expression of the *scyA* gene within pHG101 was sufficient to significantly elevate the nitrite resistance of the Δ*cyd* strain, it failed to elicit any noticeable augment in the nitrite resistance in the wild type (Fig.2A). To validate the overproduction of ScyA, western blotting was performed with antibodies against ScyA. As shown in Figure2B, amounts of ScyA from pHG101-P_*scyA*_-*scyA* in both WT and Δ*cyd* strains were similar, approximately 12-fold relative to those produced by these strains carrying the empty vector. To test whether the heme *c* of ScyA is essential to this effect, we replaced the 35th Cys residue of the heme-binding site CXX*C*H (CTV*C*H in ScyA) with a Ser, resulting in CTV*S*H. ScyA^C35S^ failed to enhance nitrite resistance of the Δ*cyd* strain, confirming that the observed effect depends on the heme *c* cofactor (Fig.2A).

In *S. oneidensis*, two other soluble *c*-type cytochromes are also among the most abundant, FccA and CctA (SO2727, small tetraheme cytochrome *c*, also known as STC) (Meyer et al. 2004; Rosenbaum et al. 2012). Both proteins are capable of mediating electron transfer from CymA to outer membrane terminal reductase MtrA (Fonseca et al. 2013). To test whether the overproduction of these two *c*-type cytochromes has a similar effect as ScyA, we repeated the experiments with their coding genes on the same overexpression vectors. However, nitrite resistance of the Δ*cyd* strain carrying the overexpression vectors remained the same (data not shown). These data, collectively, conclude that increased nitrite resistance of the Δ*cyd* strain is specifically due to the overproduction of ScyA.

Under aerobic conditions, *S. oneidensis* cells are able to convert toxic nitrite to nonharmful ammonium using periplasmic nitrite reductase NrfA (Dong et al. 2012; Zhang et al. 2013). Therefore, it is possible that the increased nitrite resistance of the Δ*cyd* strain resulting from overproduction of ScyA may be due to the fast removal of nitrite. To test this possibility, we constructed a Δ*cyd*Δ*nrfA* double mutant and compared to the Δ*cyd* strain for the resistance to nitrite when the *scyA* gene was overexpressed. As shown in Figure2A, under the circumstance of overproduction of ScyA, the Δ*cyd*Δ*nrfA* and Δ*cyd* strains displayed similar levels of the resistance to nitrite, indicating that the increased nitrite resistance of the Δ*cyd* strain conferred by overproduced ScyA is unrelated to nitrite reduction.

### ScyA is functionally linked to the cbb_3_-HCO but unlikely serves as the direct electron donor for the oxidase

**Figure 3:**
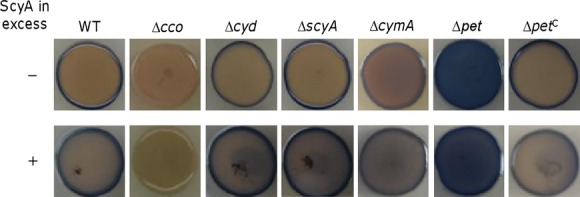
Effect of overproduction of ScyA on activities of the cytochrome *c* oxidase in test strains by the Nadi assay. The method is based on the rapid formation of indophenol blue from colorless a-naphthol catalyzed by cytochrome *c* oxidase, using *N′*,*N′*-dimethyl-p-phenylenediamine monohydrochloride as an exogenous electron donor. Nadi-positive and -negative strains were photographed 1 and 30 min after the reaction, respectively. The wild-type and Δ*cco* strains serve as positive and negative controls. Mutants showing a distinct phenotype have been successfully complemented previously. Strains without overproducing ScyA contain the empty vector. Δ*pet*^C^ represents the mutant carrying a copy of *pet* which is integrated for complementation.

In *S. oneidensis*, the *cbb*_3_-HCO is the predominant system to support growth under aerobic and microaerobic conditions and the *bd* oxidase primarily functions to facilitate survival of cells through a wide variety of stresses, nitrite in particular (Fu et al. 2013; Zhou et al. 2013; Le Laz et al. 2014). Given that the *cbb*_3_-HCO is the only oxidase present in the Δ*cyd* strain to support growth and ScyA has been proposed to be an electron donor to terminal oxidases (Meyer et al. 2004), we therefore hypothesized that ScyA in excess may enhance the activity of the oxidase, allowing growth in the presence of nitrite. The activity of the *cbb*_3_-HCO was visualized using the Nadi plate assay, which specifically detects cytochrome *c* oxidase-dependent respiration (Marrs and Gest 1973). We have previously shown that the *cbb*_3_-HCO is the only enzyme reacting with the Nadi reagents in *S. oneidensis* (Zhou et al. 2013). Consistently, the wild-type and Δ*cyd* colonies generated a blue ring in less than 1 min (Fig.3). As a negative control, a Δ*cco* strain, which lacks the *cbb*_3_-HCO, could not generate a faint blue coloration in an incubation of 20 min. The *cbb*_3_-HCO activity in both the wild-type and Δ*cyd* strains with overproduction of ScyA increased only modestly. This appears to be surprising given its significant effect on the resistance of the Δ*cyd* strain to nitrite. Nevertheless, the results suggest that ScyA is functionally relevant to but unlikely serves as an exclusive electron donor for the *cbb*_3_-HCO.

In *S. oneidensis*, both the cytochrome *bc*_1_ complex, encoded by the *pet* operon comprising the *petABC* genes, and CymA can mediate electron transport from the quinol (Q) pool to the *cbb*_3_-HCO (Fu et al. 2014). The *bc*_1_ complex is predicted to be the dominant electron donor for the *cbb*_3_-HCO because loss of the *bc*_1_ complex and the oxidase results in similar growth defect, whereas the impacts of loss of CymA on aerobic growth are rather modest (Zhou et al. 2013; Fu et al. 2014). Indeed, the Nadi assay revealed that the strain lacking CymA was comparable from the wild type, confirming that the electron transfer capacity of CymA to the *cbb*_3_-HCO is negligible when the *bc*_1_ complex is present (Fig.3). Similarly, the wild-type and Δ*cymA* strains were indistinguishable under the conditions when ScyA was overproduced. In contrast, the loss of the *bc*_1_ complex drastically increased the activity of the *cbb*_3_-HCO, with the whole colony turning blue in 1 min. Although all of these mutants have been confirmed by successful complementation in our previous studies, the striking phenotype prompted us to further validate the mutation. A copy of the *pet* operon was cloned into an integrative vector (Fu et al. 2013), which allows a single-copy complementation without antibiotic interference. The *cbb*_3_-HCO hyperactive phenotype of the Δ*pet* strain was fully corrected when this copy of the operon was expressed *in trans* (Fig.3), indicating that the phenotype observed from the Δ*pet* strain was due to the loss of the *bc*_1_ complex. The effect of overproduced ScyA in the Δ*pet* strain on the activity of the *cbb*_3_-HCO was not observed, because of the hyperactivity of the oxidase. Although further investigations are needed, we speculate that the *cbb*_3_-HCO in the strain missing the *bc*_1_ complex is idle, ready to react to the electron donor provided in the Nadi reagents (a model is provided in the discussion).

### Increased production of the cbb_3_ oxidase has a modest effect on nitrite resistance of the Δcyd strain

**Figure 4:**
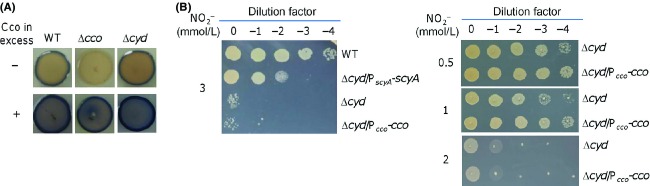
Cco in excess modestly increases nitrite resistance. The assays were repeated at least three times and similar results were obtained. (A) Effect of overproduction of the *cbb*_3_-HCO on its activity by the Nadi assay. Strains without overproducing Cco contain the empty vector. (B) Effect of overproduction of the *cbb*_3_-HCO on the nitrite resistance of the Δ*cyd* strain in the presence of nitrite of various concentrations. HCO, heme–copper oxidases.

Our results presented thus far suggest that the *cbb*_3_-HCO plays a critical role in nitrite resistance of the Δ*cyd* strain in the presence of overabundant ScyA. In an attempt to offer direct evidence, we tested whether or not the *cco* operon in multiple copies from pHG101-P_*cco*_-*cco* is able to elevate the nitrite resistance of the Δ*cyd* strain. Overproduction of the *cbb*_3_-HCO from pHG101-P_*cco*_-*cco* was evidenced by substantially increased cytochrome *c* oxidase activity using the Nadi assay (Fig.4A). In the wild-type, Δ*cco*, and Δ*cyd* strains with pHG101-P_*cco*_-*cco*, the *cbb*_3_-HCO activities were significantly higher than those in the same strains without (Fig.4A). In the case of nitrite resistance, however, no apparent increase was observed from the Δ*cyd* strain with the overproduced *cbb*_3_-HCO in the presence of 3 mmol/L nitrite (Fig.4B). We reasoned that this was likely caused by that nitrite used in the experiment overwhelmed the overproduction of the oxidase. Therefore, we retested the effect of the *cbb*_3_-HCO in overabundance on nitrite resistance of the Δ*cyd* strain with nitrite of varying concentrations. We found that nitrite at approximately 1 mmol/L but not at higher concentrations was able to elicit a difference in the resistance between cells overexpressing the oxidase or not (Fig.4B), suggesting that the amount of the *cbb*_3_-HCO is a factor in determination of the nitrite resistance of the Δ*cyd* strain.

### ScyA in excess does not affect the production of the cbb_3_-HCO

**Figure 5:**
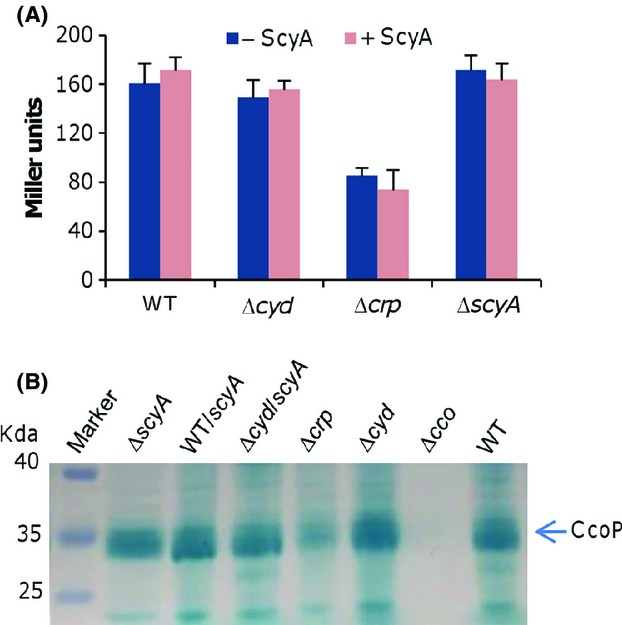
ScyA in excess affects the activity but not amount of the *cbb*_3_-HCO. (A) The activities of the *cco* promoter under indicated conditions determined by the integrative *lacZ* reporter. Error bars represent the SD of at least three independent experiments. (B) Production levels of the *cbb*_3_-HCO revealed by heme staining of CcoP. Proteins (10 *μ*g per lane) extracted from indicated strains were separated on SDS-PAGE and analyzed by heme staining. The assays were repeated at least three times and similar results were obtained. HCO, heme–copper oxidases; SDS-PAGE, sodiumdodecyl sulphate polyacrylamide gel electrophoresis.

The overabundant *cbb*_3_-HCO in the experiment described above is insufficient to confer nitrite resistance to levels comparable to those resulting from ScyA in excess, explaining why we did not obtain any suppressors of the Δ*cyd* strain in proximity of the *cco* operon during the transposon screening. Nonetheless, whether or not the increased *cbb*_3_-HCO activity in the presence of excess ScyA results from overabundance of the enzyme merits further investigation. To this end, we assessed the expression of the *cco* operon using the integrative *lacZ* reporter in strains overproducing ScyA. The promoter of the *cco* operon, which was defined before (Zhou et al. 2013), was cloned into pHGEI01 and the resulting vector was introduced into both the wild-type and Δ*cyd* strains. A strain (Δ*crp*) lacking the *crp* gene, whose product mediates the expression of the *cco* and *cyd* operons in *S. oneidensis* (Fu et al. 2013; Zhou et al. 2013), was included in the analysis as a control. After integration and removal of the antibiotic marker, pHG101-P_*scyA*_-*scyA* was introduced. As shown in Figure5A, the activities of P_*cco*_ were comparable in the mid-log phase cells of all test strains except for the Δ*crp* strain. The compromised P_*cco*_ activity resulting from the loss of Crp was consistent with the finding in our previous report (Zhou et al. 2013). To confirm, we extracted proteins from these samples, separated them by SDS-PAGE, and stained for covalently bound heme with 3,3′,5,5′-tetramethylbenzidine (TMBZ). Similarly, the amounts of CcoP, a *c*-type cytochrome subunit of the *cbb*_3_-HCO, were comparable in all samples except for the Δ*crp* strain (Fig.5B). These data conclude that the abundance of ScyA has little effect on the production of the *cbb*_3_-HCO.

### Effect of overproduced ScyA depends on a complete electron transport chain to cbb_3_-HCO

**Figure 6:**
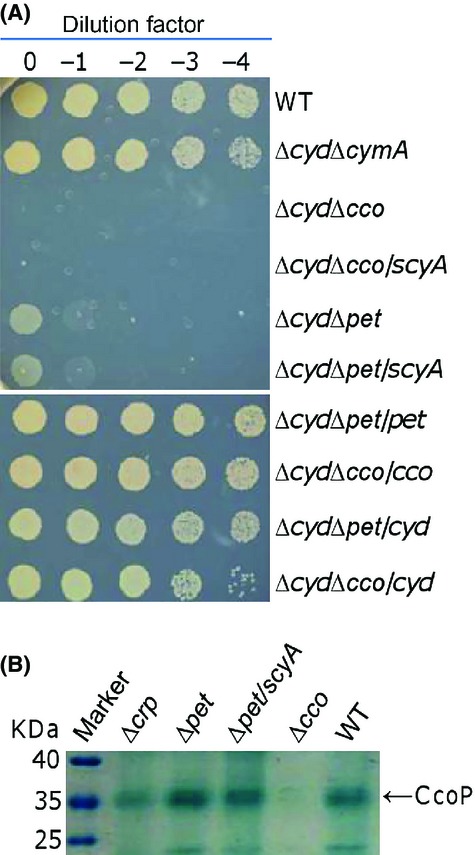
Function of ScyA in excess relies on a complete electron transfer to the *cbb*_3_-HCO. The assays were repeated at least three times and similar results were obtained. (A) Effect of overproduction of ScyA on the nitrite resistance of the Δ*cydΔcco* and Δ*cydΔpet* strain in the presence of 3 mmol/L nitrite. (B) Effect of overproduction of ScyA on production levels of the *cbb*_3_-HCO revealed by heme staining of CcoP. Proteins (10 *μ*g per lane) extracted from indicated strains were separated on SDS-PAGE and analyzed by heme staining. HCO, heme–copper oxidases; SDS-PAGE, sodiumdodecyl sulphate polyacrylamide gel electrophoresis.

Although ScyA unlikely serves as the predominant electron donor for the *cbb*_3_-HCO, it is functionally linked to the oxidase. To further confirm this, we intended to address whether the observed effect of overproduced ScyA requires functional *cbb*_3_-HCO. Unfortunately, the effect of overproduced ScyA on the activity of the *cbb*_3_-HCO in the Δ*pet* strain could not be examined using the Nadi assay because of the hyperactivity of the oxidase (Fig.3A). We then utilized an alternative method to test this. Attempts were made to generate double mutants lacking the *bd* oxidase and either the *bc*_1_ complex or CymA. While a Δ*cyd*Δ*cymA* strain was obtained smoothly, we failed to delete the *cyd* and *pet* operons under aerobic conditions. Given that the *cbb*_3_-HCO and the *bd* oxidase are a combination of synthetic lethal, these observations suggest that CymA alone is insufficient to ensure the function of the *cbb*_3_-HCO (Zhou et al. 2013). By performing mutagenesis under anaerobic conditions, we successfully obtained a strain (Δ*cyd*Δ*pet*) lacking both the *bc*_1_ complex and the *bd* oxidase, which could hardly grow aerobically (Fig.6A). Expression of either system *in trans* in the double mutant enabled growth under aerobic conditions, confirming that the inability to grow with oxygen is due to intended mutations. In line with the proposed role of the *bc*_1_ complex (Dibrova et al. 2013), these results conclude that the *cbb*_3_-HCO is, largely but not exclusively, dependent on the *bc*_1_ complex for electrons from the quinol pool. The residual activity of the *cbb*_3_-HCO in the absence of the *bc*_1_ complex is probably from CymA, which has been recently shown to overlap with the *bc*_1_ complex to some extent in respiration of nitrate and nitrite (Fu et al. 2014). ScyA in excess failed to rescue the aerobic growth of the Δ*cyd*Δ*pet* strain, a scenario also observed from the Δ*cyd*Δ*cco* strain. To rule out a possibility that loss of the *bc*_1_ complex prevents the production of the *cbb*_3_-HCO, cellular levels of the oxidase were examined. As shown in Figure6B, amounts of the *cbb*_3_-HCO in strains containing the *bc*_1_ complex or not were comparable. These data, collectively, indicate that ScyA in overabundance is unable to exert its effect unless the electron transfer to the *cbb*_3_-HCO is in place.

### ScyA in excess alleviates inhibition of nitrite on the cbb_3_-HCO

As the overabundance of the *cbb*_3_-HCO is not sufficient to confer the Δ*cyd* strain nitrite resistance comparable to that resulting from ScyA in excess, we hypothesized that overproduced ScyA may increase resistance of the *cbb*_3_-HCO to nitrite. To investigate this possibility, we assessed the effects of ScyA in overabundance on the half-maximal inhibitory concentration of the *cbb*_3_-HCO and the *bd* oxidase for nitrite (IC_50_).

**Figure 7:**
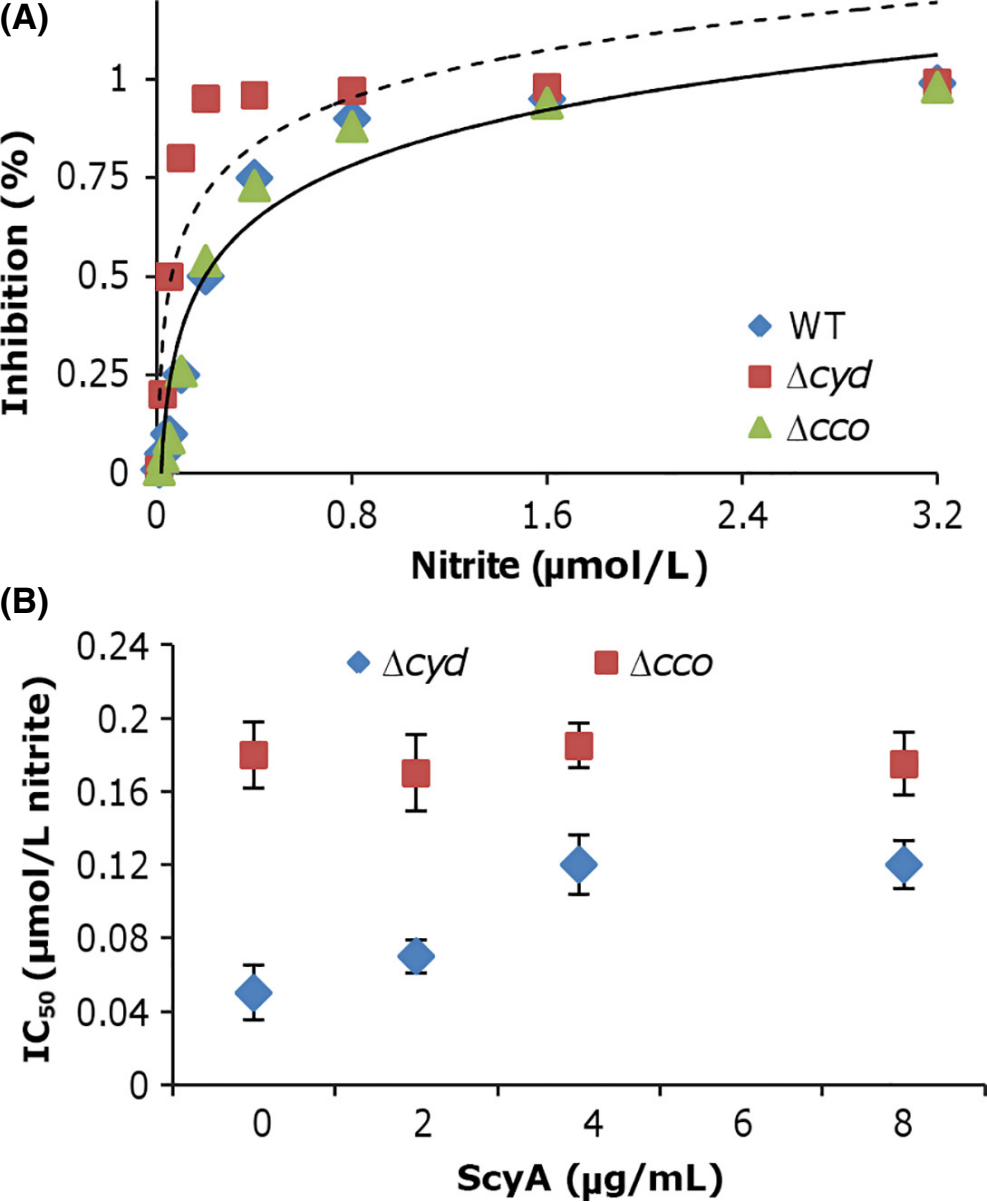
ScyA in excess improves the nitrite resistance of the *cbb*_3_-HCO. Error bars represent the SD of at least three independent experiments. A. Nitrite sensitivity of the cytochrome *bd* and *cbb*_3_-HCO. Respiration rates of membranes were measured in the presence of nitrite of various concentrations. The dash line was derived from the Δ*cyd* strain and the solid lines were derived from WT and Δ*cco* strains, which were undistinguishable. B. Effect of overproduction of ScyA on nitrite IC_50_ values of the cytochrome *bd* and *cbb*_3_-HCO. The half-maximal inhibitory concentrations for nitrite were measured as described in Experimental procedures. HCO, heme–copper oxidases; WT, wild type; IC, ion chromatography.

Membrane preparations of *S. oneidensis* strains expressing only one of the terminal oxidases were used to measure oxygen reduction with ubiquinol-1 for the *bd* oxidase or with a combination of ascorbate and TMPD for the *cbb*_3_-HCO (Mason et al. 2009; Chen et al. 2014; Xie et al. 2014). At ∼70 *μ*mol/L O_2_, the IC_50_ value of the *S. oneidensis bd* oxidase was approximately 0.18 *μ*mol/L, whereas the IC_50_ value for the *cbb*_3_-HCO was about 0.05 *μ*mol/L (Fig.7A), consistent with the observation that the Δ*cyd* strain is more sensitive to nitrite than the Δ*cco* strain. No difference in the IC_50_ values between the wild-type and the Δ*cco* strain was observed, further validating that the *bd* oxidase is the enzyme that dictates nitrite resistance. Unfortunately, membrane preparations of *S. oneidensis* strains overexpressing ScyA did not show any effect on the IC_50_ values of the Δ*cyd* or Δ*cco* strain (data not shown). We suspected that this may be due to the loss of ScyA during the preparation for membranes. To circumvent this problem, we added a His_6_-tag after the C-terminus of ScyA and expressed the fusion protein in *S. oneidensis* for purification (Fig. S2). When in excess the His_6_-tag ScyA was able to elevate the nitrite resistance of the Δ*cyd* strain to levels obtained from ScyA (Fig. S1), indicating that the tagged protein is biologically active. With purified His_6_-ScyA, the IC_50_ values of membrane preparations from the Δ*cyd* and Δ*cco* strains were measured (Fig.7B). While the addition of His_6_-ScyA had no effect of the IC_50_ values for the *bd* oxidase (Δ*cco*), it increased those for the *cbb*_3_-HCO (Δ*cyd*) proportionally with 4 *μ*g/mL or less and further improvement was not observed with more proteins. Notably, the IC_50_ values for the *bd* oxidase exceeded those for the *cbb*_3_-HCO at all protein concentrations. These data, all together, indicate that ScyA improves nitrite resistance of *S. oneidensis* by reducing the sensitivity of the *cbb*_3_-HCO to nitrite.

## Discussion

The purpose of this study was to identify proteins other than the *bd* oxidase that facilitate aerobic growth of *S. oneidensis* in the presence of nitrite. We have shown previously that the *bd* oxidase is the primary force conferring this microorganism resistance to nitrite (Fu et al. 2013), a scenario similar to that the *E. coli* counterpart does on NO resistance (Mason et al. 2009). *S. oneidensis* is able to reduce nitrite to ammonia under aerobic condition, leading to detoxification (Dong et al. 2012; Zhang et al. 2013; Fu et al. 2014). However, there is a caveat: cells have to be in the stationary phase, even with the forced production of periplasmic nitrite reductase NrfA (Zhang et al. 2013). Thus, mutations that enable cells to respire on nitrite before the stationary phase are likely to improve growth with nitrite. It is also possible that the loss of some porins and/or transporters by which nitrite enters the periplasm would make a difference by slowing nitrite influx (Song and Niederweis 2012). Moreover, there may be an alternative oxidase, similar to that found in *Vibrio fischeri* which is highly resistant to NO (Dunn et al. 2010), encoded by a gene hidden in the genome as up to 40% genes are for hypothetical proteins (Heidelberg et al. 2002).

Given that multiple means for enhancing nitrite resistance possibly exist, we anticipated that we would identify a few. To our surprise, only ScyA, a monoheme cytochrome *c*_5_ that is suggested to be one of the most abundant periplasmic proteins found in cells under anaerobic conditions (Tsapin et al. 2001; Meyer et al. 2004; Fonseca et al. 2013), was caught. Such a finding is enabled by a new mariner-based transposon vector, developed in this study, which carries a highly active promoter for the *ompS38* gene within the transposable segment. In *S. oneidensis*, OmpS38 is evidently one of the most abundance porins although its expression is barely affected by the EnvZ-OmpR two-component regulatory system (Yuan et al. 2011). Interestingly, production of the protein is upregulated under anaerobic conditions (Maier and Myers 2004). These features, altogether, render the vector suitability for screening for cryptic or quiescent operons in addition to knockout of active ones.

Despite intensive studies of *S. oneidensis c*-type cytochromes as the foundation for its respiratory versatility, we are only just beginning to uncover the molecular mechanisms underlying their physiological roles. This is particularly true for soluble *c*-type cytochromes as they are featured by a large number, up to 28 (Meyer et al. 2004), and diffusibility in the periplasm, the latter providing opportunities to interact with many similarly soluble and/or membrane-bound proteins. Two such proteins which are also among the most abundant, FccA and CctA serve as good examples. FccA is a soluble tetraheme flavocytochrome *c* fumarate reductase carrying heme cores that are distinct from those observed in well-studied tetraheme cytochrome *c*_3_ of *Desulfovibrio* (Leys et al. 1999; Taylor et al. 1999). In addition to catalyzing fumarate reduction, FccA mediates electron transfer from CymA to MtrA of the metal reduction complex MtrABC (Schuetz et al. 2009; Fonseca et al. 2013). Moreover, FccA, by binding to CymA to form a redox complex, regulates the direction of catalysis and electron transfer of the latter (McMillan et al. 2013). Similar to FccA, CctA is also able to interact with both CymA and MtrA, playing an important role in reduction of metal oxide particles (Gordon et al. 2000; Leys et al. 2002; Fonseca et al. 2013).

ScyA, like FccA and CctA, is also able to interact with CymA and proposed to be the only periplasmic electron donor for CcpA, a diheme *c*-type cytochrome peroxidase (Schütz et al. 2011; Fonseca et al. 2013). Here, we showed that ScyA in excess enhances the activity of the *cbb*_3_-HCO. Multiple insertions were mapped into the promoter region of the *scyA* gene, indicating that the random mutant pool is sufficiently large to completely cover all genes and intergenic sequences in between. Single catch implies that ScyA is likely the only one which in excess is able to improve nitrite resistance of the *cbb*_3_-HCO to the observed extent.

It is generally accepted that cytochrome *c* oxidases do not directly oxidize the quinol pool but via soluble cytochrome *c* electron shuttles, which are functionally essential to the enzymes (Ekici et al. 2012). Although proposed to be an electron donor to cytochrome *c* oxidases (Meyer et al. 2004), ScyA is unlikely to be predominant one based on the drastic difference in the Nadi staining between strains missing the *bc*_1_ complex and ScyA. Given that *S. oneidensis* is equipped with many cytochromes of similar redox potentials and the electron transport processes mediated by *c*-type cytochrome are suggested to be unspecific (Firer-Sherwood et al. 2008; Coursolle and Gralnick 2012; Fonseca et al. 2013), we would expect that more than one cytochrome *c* are able to serve as the electron donor for the *cbb*_3_-HCO. Moreover, the *bc*_1_ complex and CymA overlap each other in mediating electron transfer from the quinone pool to terminal reductases (HCOs actually are oxygen reductases) to some extent and are exchangeable under certain conditions, further broadening the scope of such *c*-type cytochromes (Fu et al. 2014). Thus, it is possible that multiple small *c*-type cytochromes may mediate electron transfer from the *bc*_1_ complex to the *cbb*_3_-HCO, a scenario reported in *Rhodobacter sphaeroides* before (Daldal et al. 2001).

Based on our data, we propose two mechanisms that underlie enhanced nitrite resistance of the *cbb*_3_-HCO in the presence of overabundant ScyA. One involves with electron transfer. As ScyA is a minor player in transferring electrons from the *bc*_1_ complex/CymA to the *cbb*_3_-HCO, its loss does not significantly compromise the activity of the *cbb*_3_-HCO. This is evident with the phenotypes of the *scyA* mutant, with respect to aerobic growth, nitrite susceptibility, and the activity revealed by the Nadi assay. When in excess, ScyA may be able to increase the electron turnover rate of the *cbb*_3_-HCO, resulting in the augment of the nitrite resistance. This notion gains support clearly from in vivo data. Perhaps more importantly, it is also in line with in vitro results, at least to some extent. The in vitro experiment was conducted with NADH, a condition under which ScyA likely exists in its reduced form. Hence, electron transfer may occur from ScyA to the *cbb*_3_-HCO. The other involves direct interaction with the *cbb*_3_-HCO. The *E. coli* cytochrome *bd* oxidase, which confers NO resistance to *E. coli*, has a NO dissociation rate faster than both the cytochrome *bo* oxidase and *aa*_3_-HCO(Mason et al. 2009). Given that the in vitro data presented in Figure7 resemble those reported in the *E. coli* study, it is therefore possible that ScyA in excess helps alter the biochemical properties of the *cbb*_3_-HCO, leading to its increased resistance to nitrite. How these intricate processes are spatially and temporally coordinated are currently under our investigation.

**Figure 8:**
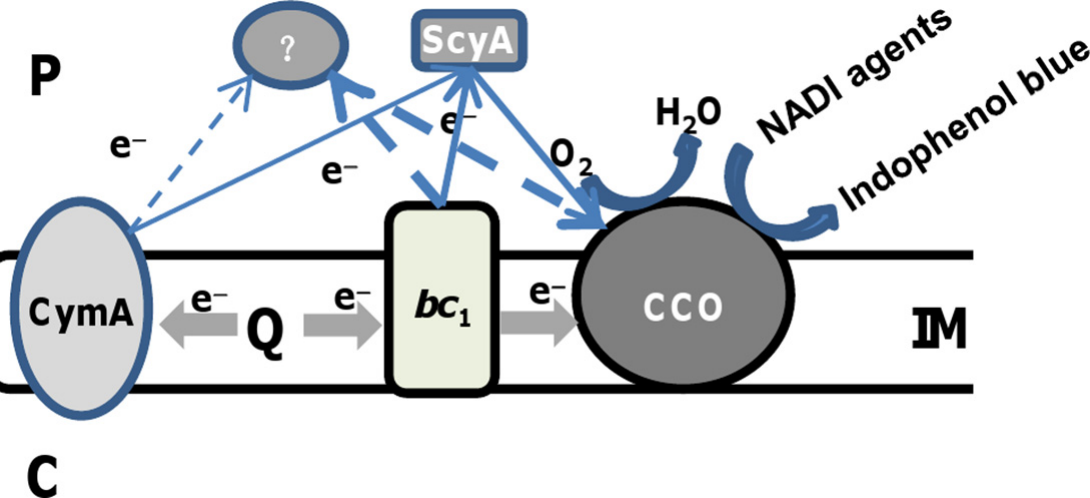
Model: electron transfer to the *cbb*_3_-HCO in *Shewanella oneidensis*. The cytochrome *bc*_1_ complex may transfer electrons to the *cbb*_3_-HCO directly or more likely via an undetermined cytochrome *c* as the major mediator and ScyA as the minor, allowing oxygen reduction. Contribution from CymA as the electron source is extremely limited. According to the Nadi reaction (*α*-naphthol + DMPD + O_2_ → Indophenol Blue + H_2_O), oxygen is required for both oxygen respiration and the Nadi reaction. Solid and dash lines represent established and suggested electron pathways, respectively. C, IM, and P represent cytoplasm, inner-membrane, and periplasm, respectively.

Our results presented here and before demonstrated that the cytochrome *bc*_1_ complex is essential to the *cbb*_3_-HCO for reducing oxygen (Zhou et al. 2013; Fu et al. 2014). So, why does the strain devoid of the complex exhibit an enhanced activity of the *cbb*_3_-HCO? We suggest a mechanism illustrated in Figure8. In *S. oneidensis*, there are two ways to supply electrons to the *cbb*_3_-HCO for oxygen respiration: the major one is through the *bc*_1_ complex, which may couple with a yet unidentified cytochrome *c*, and the minor one is mediated by ScyA. Both CymA and the *bc*_1_ complex may serve as the electron donors for these *c*-type cytochromes as ScyA has been shown to be able to interact with CymA (Coursolle and Gralnick 2010; Schütz et al. 2011). However, contribution from CymA as the electron source is negligible. Neither of these two electron mediators is required for the Nadi assay: *α*-naphthol + *N′,N′*-dimethyl-p-phenylenediamine (DMPD) + O_2_ → Indophenol Blue + H_2_O because DMPD serves as the electron donor (Marrs and Gest 1973). However, oxygen is the substrate for both the Nadi reaction and oxygen respiration, which requires electrons from the *bc*_1_ complex and/or cytochrome *c* electron mediators. As a consequence, the two processes compete with each other for oxygen. When electrons are no longer delivered to the *cbb*_3_-HCO, the enzyme exclusively reacts with the Nadi reagents, exhibiting the highest activities. It is worth mentioning that it is still uncertain of whether or not there exists a yet unidentified cytochrome *c* mediating electron transfer from the cytochrome *bc*_1_ complex to the *cbb*_3_-HCO. Efforts to test this are underway.

## Supporting Information

**Figure S1.** Characteristics of recombinant ScyA. (A) SDS-PAGE analysis of purified recombinant ScyA by heme-staining. ScyA-His_6_ was extracted from the *S. oneidensis* wild-type carrying pHGE-ScyA-His_6_. M represents protein marker. (B) Functional analysis of recombinant ScyA and mutant proteins.
