# Supplementary material for: Regulation of nitrite resistance of the cytochrome cbb3 oxidase by cytochrome c ScyA in Shewanella oneidensis
Source: Microbiologyopen. 2014 Nov 22;4(1):84–99. doi: 10.1002/mbo3.224 (PMC4335978; doi:10.1002/mbo3.224)
Supplement: Supplementary file 1 [file mbo30004-0084-sd1.pdf]

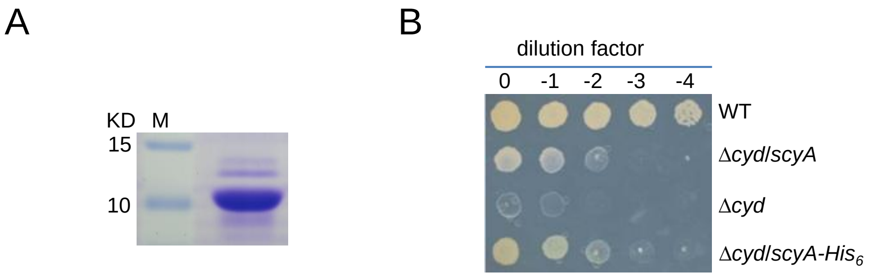

Fig. S1. Characteristics of recombinant ScyA.

A. SDS-PAGE analysis of purified recombinant ScyA by heme-staining. ScyA-His$_6$ was extracted from the *S. oneidensis* wild-type carrying pHGE-ScyA-His$_6$. M represents protein marker.

B. Functional analysis of recombinant ScyA and mutant proteins.

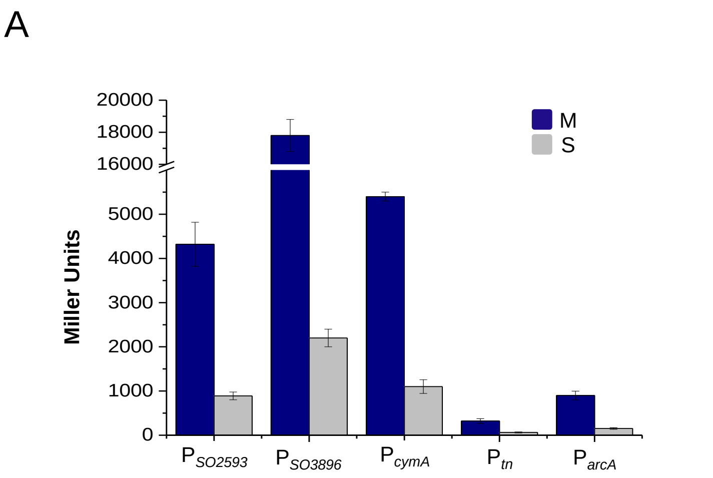

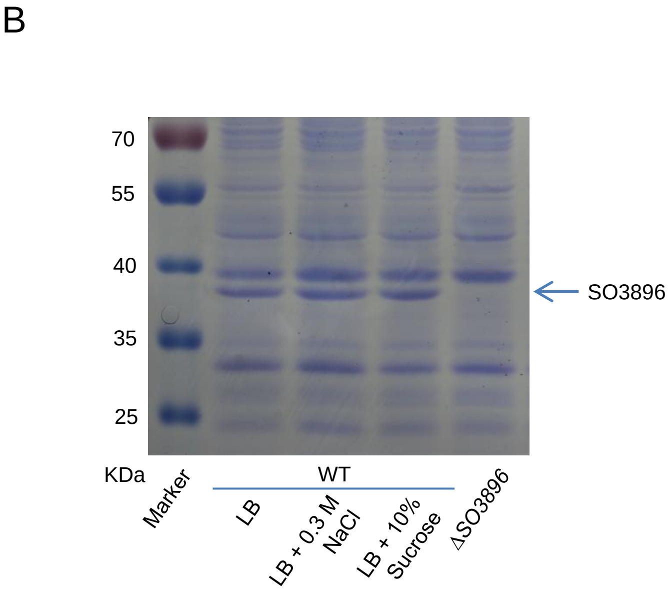

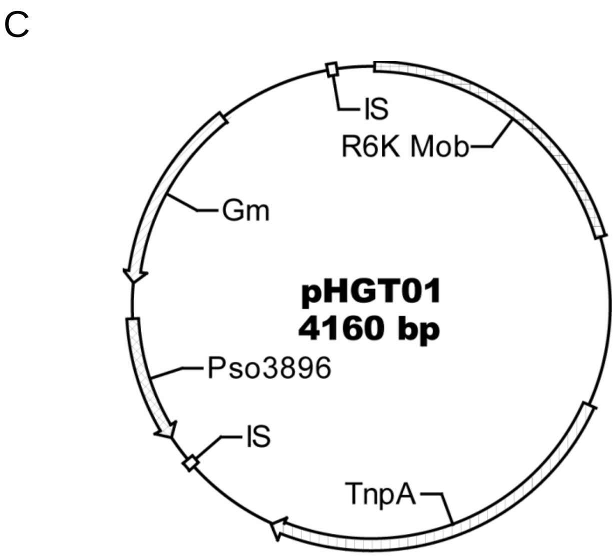

Fig. S2. Development of a new mariner-based transposon vector pHGT01

A. Screening for extremely strong promoters. The activities of the P_{SO2503}, P_{SO3896}, P_{cymA}, P_{tn}, and P_{arcA} revealed by *lacZ* reporters. Cells of mid-log phase were sampled for the assay. Both multi-copy (M) and single-copy (S) systems were used. The values are the mean ± S.D. (error bars) (n = 5).

B. SO3896 in the outer membrane in the presence or absence of osmotic agents. Wild-type (WT) cells were grown overnight in LB and subcultured 1:200 in fresh medium in the presence or absence of indicated agents. SO3896 protein band is indicated. Experiments were repeated at least three times and similar results were obtained.

C. The vector was created by replacing transposon-unrelated DNA sequence with the *R6K* replicon and the *mob* gene and inserting outward P_{SO3896} inside IS elements.
